# Pleiotropic genetic association analysis with multiple phenotypes using multivariate response best-subset selection

**DOI:** 10.1186/s12864-023-09820-5

**Published:** 2023-12-11

**Authors:** Hongping Guo, Tong Li, Zixuan Wang

**Affiliations:** 1 School of Mathematics and Statistics, Hubei Normal University, Huangshi, 435002 People’s Republic of China; 2 School of Mathematics and Statistics, South-Central Minzu University, Wuhan, 430074 People’s Republic of China

**Keywords:** Pleiotropy, Association analysis, Multiple phenotypes, Response variable selection, Best-subset, 0-1 integer optimization

## Abstract

Genetic pleiotropy refers to the simultaneous association of a gene with multiple phenotypes. It is widely distributed in the whole genome and can help to understand the common genetic mechanism of diseases or traits. In this study, a multivariate response best-subset selection (MRBSS) model based pleiotropic association analysis method is proposed. Different from the traditional genetic association model, the high-dimensional genotypic data are viewed as response variables while the multiple phenotypic data as predictor variables. Moreover, the response best-subset selection procedure is converted into an 0-1 integer optimization problem by introducing a separation parameter and a tuning parameter. Furthermore, the model parameters are estimated by using the curve search under the modified Bayesian information criterion. Simulation experiments show that the proposed method MRBSS remarkably reduces the computational time, obtains higher statistical power under most of the considered scenarios, and controls the type I error rate at a low level. The application studies in the datasets of maize yield traits and pig lipid traits further verifies the effectiveness.

**Supplementary Information:**

The online version contains supplementary material available at 10.1186/s12864-023-09820-5.

## Introduction

Genome-wide association study (GWAS) has proven to be a potent tool for elucidating the genetic loci implicated in complex diseases or phenotypes [1]. To date, tens of thousands of disease-associated single nucleotide polymorphisms (SNPs) have been identified by scanning SNP one by one for each disease. However, the single-locus GWAS methods are plagued by several limitations, including the weak marginal effects, the disregard for genetic locus interactions, and the need for stringent multiple testing corrections. To overcome these shortcomings, multi-locus GWAS methods considering the joint effect of SNPs have been proposed. Since the “large *p* (number of SNPs), small *n* (sample size)” problem, many efforts focus on developing multi-locus models based on regularization or penalized regression techniques, such as the least absolute shrinkage and selection operator (LASSO) [2], Bayesian LASSO [3] and Elastic Net [4]. Moreover, Segura et al. [5] introduced a multi-locus mixed effect model approach employing a stepwise regression framework that combines forward selection and backward elimination. Wen et al. [6] proposed a two-stage strategy method wherein the first stage involves the selection of potential SNPs using a single-locus approach, followed by testing the selected SNPs in a multi-locus model.

With the deepening of GWAS and epidemiological studies, more and more evidence suggests the widespread existence of genetic pleiotropy, which refers to a phenomenon that a single gene is simultaneously associated with multiple phenotypes [7, 8]. According to the GWAS-Catalog, a compilation of published genome-wide association studies by the National Human Genome Research Institute (NHGRI) in the United States, approximately 16.9% of genes exhibit across the genome [9]. For example, the gene C11orf30, implicated in the regulation of total serum IgE levels, has been linked not only to asthma but also to various allergic disorders such as hay fever and eczema, potentially through cytokine release modulation [10]. Considering pleiotropic effects not only provides meaningful biological interpretations but also enhances the power of genetic association analysis, a large amount of multi-trait association analysis methods have been developed in the past decade, which can be broadly classified into two categories: (1) Univariate analyses. To be specific, single-trait association analysis method is initially performed for each of the phenotypes, subsequently, a specific *P*-value combination method, such as Fisher’s method [11], weighted method [12] or Cauchy’s method [13], is employed to obtain the final aggregated *P*-value. (2) Multivariate analyses. The basic idea is to conduct association test between the candidate SNP and multiple phenotypes simultaneously. This kind of approaches encompasses two representative methodologies: model-based methods and dimension reduction methods. Model-based methods mainly include linear mixed model method and Bayesian model method for continuous data [14, 15], proportional odds model method for ordered data [16], and non-parametric model method [17]. On the other hand, dimension reduction methods, including principal component analysis [18], canonical correlation analysis [19], and hierarchical clustering analysis [20], are specifically designed to reduce the dimensionality of the phenotypes. Subsequently, these low-dimension phenotypes are utilized to investigate the association with the candidate SNP.

The aforementioned multi-trait methods implement association test for a single variant or gene at a time, there are only a few multi-trait multi-locus methods to detect genetic pleiotropy to our current knowledge. For example, some distance regression model methods have been proposed to perform association test based on the similarity matrices of genotype and phenotype [21–24]. Samuel et al. [25] developed a multi-trait, multi-locus stepwise model selection procedure that proves to be valuable in scenarios where phenotypes are influenced by both pleiotropic and non-pleiotropic quantitative trait nucleotides. Bottolo et al. [26] proposed a Bayesian variable selection to perform polygenic association with multiple phenotypes, and exploited parallel processing framework for fully multivariate modelling of groups of correlated phenotypes at the genome-wide scale. In practice, many of SNPs do not take effect on any of the phenotypes. If all of the SNPs are included in the genetic models, it will not only increase the complexity and computational burden of the models, but also hinder the estimation of regression coefficient and the final variable selection. Consequently, removing the irrelevant SNPs (i.e., those not related to any phenotype) is very important for deciphering the pleiotropic genetic associations between SNPs and multiple phenotypes.

In this study, we apply the multivariate response best-subset selection method (MRBSS) to perform pleiotropic genetic association analysis. MRBSS method is recently proposed by Hu et al. [27], it can perform response variable selection and regression coefficient estimation simultaneously for multivariate regression model with high-dimensional response variables. Different from the traditional genetic association model, we view the high-dimensional genotypic data as response variables while the multiple phenotypic data as predictor variables in the multivariate response variable selection genetic model. Then, we convert the response best-subset selection procedure into an 0-1 integer optimization problem by introducing a separation parameter and a tuning parameter. Finally, we estimate the model parameters by using the curve search under the modified Bayesian information criterion.

The rest of paper is organised as follows. “Proposed pleiotropic genetic association analysis method” section introduces the proposed pleiotropic genetic association analysis method MRBSS. “Simulation studies” section compares the performance of other two methods in terms of statistical power, type I error rate and computational time through simulated experiments. “Real data applications” section analyzes the data from two real datasets, namely maize yield-related phenotypes and pig lipid phenotypes. Finally, “Discussion” section gives some discussions.

## Proposed pleiotropic genetic association analysis method

### Multivariate response variable selection genetic model

Suppose that there are *n* independent samples derived from a source population. For each sample, data of *q* SNPs and *p* phenotypes of interest are collected. Different from the traditional genetic association model, the high-dimensional genotypic data are viewed as response variables and the multiple phenotypic data as predictor variables. Then, we consider a multivariate response variable selection genetic model as follows:

1 $$\begin{aligned} Y\Delta =X\Theta +{\varepsilon }{\Delta }, \end{aligned}$$

where *Y* is an $n\times q$ SNP genotype matrix, and *X* is an $n\times p$ phenotype matrix adjusted for covariates such as population stratification. $\Delta =diag(\delta _1,\delta _2,\cdots ,\delta _q)$ is an $q\times q$ response subset selection matrix, whose diagonal elements are selection factors $\delta _j (j=1,2,\cdots ,q)$ with two possible values $\delta _j=0$ or 1. If $\delta _j=1$, the corresponding *j*th SNP is considered to be active (i.e., associated with at least one phenotype), otherwise, if $\delta _j=0$, the *j*th SNP is considered to be inactive (i.e., not associated with any of the phenotypes). $\Theta$ is an $p \times q$ regression coefficient matrix. $\varepsilon$ is the residual error matrix, which satisfies row independence and each row follows a multivariate normal distribution with mean 0 and variance $\Sigma$. Therefore, the mean and covariance of $\varepsilon$ are $E(\varepsilon )={\textbf {0}}$ and $Cov(\varepsilon )=I_n \otimes \Sigma$, respectively, where $\otimes$ denotes the Kronecker product. In essence, the genetic model (1) can be seen as a generalization of multivariate linear regression model $Y=X\Theta +{\varepsilon }$, which can be divided into *q* linear regression models $y_j=X\Theta _j+\varepsilon _j$, where $\Theta _j$ is an $p\times 1$ regression coefficient vector, and $\varepsilon _j$ is the *j*th column of the residual error matrix $\varepsilon$. The null hypothesis and alternative hypothesis are as follows:

$$\begin{aligned} H_0^j:{\Theta }_j=0\quad \longleftrightarrow \quad H_1^j:{\Theta }_j\not =0 \quad (j=1,2,{\cdots },q), \end{aligned}$$

Obviously, $H_0^j:{\Theta }_j=0$, which indicates the *j*th SNP has no association with any of the phenotypes, is equivalent to $\delta _j=0$ and corresponds to the active one. Conversely, $H_1^j:{\Theta }_j\not =0$, which means the *j*th SNP has association with at least one of the phenotypes, is equivalent to $\delta _j=1$ and corresponds to the inactive one.

### Response best-subset selection

In this study, we aim to find the best subset of active SNPs, so it is necessary to effectively distinguish between active and inactive SNPs. On one hand, we introduce a separation parameter $\gamma \in [0,1]$ and construct an adaptive weight matrix as follows:

2 $$\begin{aligned} W=diag\left(\left(y_1^{\top }P_X{y_1}\right)^{1+\gamma },\left(y_2^{\top }P_X{y_2}\right)^{1+\gamma },{\cdots },\left(y_q^{\top }P_X{y_q}\right)^{1+\gamma }\right), \end{aligned}$$

where $P_X=X(X^{\top }X)^{-}X^{\top }$ is the projection matrix, $^{\top }$ represents the transpose of a matrix or vector, and $^{-}$ denotes the generalized inverse. The parameter $\gamma$ describes the degree of separation between active and inactive SNPs. On the other hand, we perform penalizing the dual transformation of the selection factors, $1-\delta _1,\cdots , 1-\delta _q$. Then, we construct penalized multivariate least-squares function as follows:

3 $$\begin{aligned} Q(\Delta ,\Theta )=\frac{1}{n}||Y\Delta -X\Theta ||_F^2+\frac{1}{n}\lambda {\ \cdot \ }tr\{W(I-\Delta )\}, \end{aligned}$$

where $\lambda \in (0,\infty )$ is a tuning parameter, $||A||_F^2=tr(AA^{\top })$ represents the Frobenius norm of matrix *A*, and *tr* denotes the trace of a matrix.

To solve the response best-subset selection problem, which involves selecting the true active-SNP subset while excluding the inactive-SNP subset, we can convert it to a mixed 0-1 integer optimization problem:

4 $$\begin{aligned} \min Q(\Delta ,\Theta ). \end{aligned}$$

The solution of (4) can be expressed as:

5 $$\begin{aligned} (\hat{\Delta },\hat{\Theta })=\arg \min \left\{\frac{1}{n}||Y\Delta -X\Theta ||_F^2+\frac{1}{n}\lambda {\ \cdot \ }tr\{W(I-\Delta )\}\right\} . \end{aligned}$$

Notice that the least-squares estimate of regression coefficients can be obtained from model (1), i.e., $\hat{\Theta }=X(X^{\top }X)^{-1}X^{\top }Y\Delta$. Take $\hat{\Theta }$ into (5), and decompose the objective function into two main terms: $||Y\Delta -X\hat{\Theta }||_F^2=\sum _{j=1}^{q}{y_j^{\top }}(I-P_X){y_j}{\delta }_j$ and $tr\{W(I-\Delta )\}=\sum _{j=1}^{q}\left({y_j^{\top }}P_X{y_j}\right)^{1+\gamma }(1-{\delta }_j)$. Thus, solving the response best-subset selection problem (4) is further transformed into a pure 0-1 integer optimization problem:

6 $$\begin{aligned} H_n(\Delta )=\frac{1}{n}\sum \limits _{j=1}^{q}{y_j^{\top }}(I-P_X){y_j}{\delta }_j+\frac{1}{n}{\lambda }{\cdot }\sum \limits _{j=1}^{q}\left({y_j^{\top }}P_X{y_j}\right)^{1+\gamma }(1-{\delta }_j), \end{aligned}$$

7 $$\begin{aligned} \min H_n(\Delta ). \end{aligned}$$

where $H_n(\Delta )$ can be seen as a sum of *q* individual objective functions, that is, $H_n(\Delta )=\sum _{j=1}^{q}{H_n^j}(\delta _j)$, and the *j*th objective function can be expressed as:

8 $$\begin{aligned} {H_n^j}(\delta _j)=\frac{1}{n}{y_j^{\top }}(I-P_X){y_j}{\delta }_j+\frac{1}{n}{\lambda }{\ \cdot \ }\left({y_j^{\top }}P_X{y_j}\right)^{1+\gamma }(1-{\delta }_j). \end{aligned}$$

Obviously, $\min H_n(\Delta )$ in (7) is equivalent to $\min {H_n^j}(\delta _j)$ for $j=1,2,{\cdots },q$. If $\delta _j=0$, ${H_n^j}(\delta _j)=\frac{1}{n}{\lambda }{\cdot }\left({y_j^{\top }}P_X{y_j}\right)^{1+\gamma }$; If $\delta _j=1$, ${H_n^j}(\delta _j)=\frac{1}{n}{y_j^{\top }}(I-P_X){y_j}$. The minimizer of ${H_n^j}(\delta _j)$ is $\delta _j=1$ if the condition ${y_j^{\top }}(I-P_X){y_j}\le {\lambda }{\cdot }\left({y_j^{\top }}P_X{y_j}\right)^{1+\gamma }$ satisfied, or $\delta _j=0$ otherwise. Therefore, the solution set for the response best-subset selection problem can be defined as:

9 $$\begin{aligned} \mathscr {A}_j=\left\{y_j:{y_j^{\top }}(I-P_X){y_j}\le {\lambda }{\ \cdot \ }\left({y_j^{\top }}P_X{y_j}\right)^{1+\gamma },\gamma \in [0,1],\lambda \in (0,\infty )\right\}. \end{aligned}$$

The solution in the estimated response best-subset selection matrix $\hat{\Delta }=diag\left(\hat{\delta _1},\hat{\delta _2},{\cdots },\hat{\delta _q}\right)$ is defined as:

10 $$\begin{aligned} \hat{\delta _j}=\left\{ \begin{array}{cc} 1,\ \ {} &{} \ \ y_j\in \mathscr {A}_j,\\ 0,\ \ {} &{} \ \ other. \end{array} \right. \end{aligned}$$

### Separation and tuning parameter estimates

In the last section, we provide the solution set form for the response best-subset selection problem to find the active-SNP subset, which includes the separation parameter $\gamma$ and the tuning parameter $\lambda$. In the following, we will explain how to determine the parameter pair $(\gamma , \lambda )$. Similar to the existing literature [27], we apply the modified Bayesian information criterion (BIC) to estimate the aforementioned parameter pair. The specific expression is as follows:

11 $$\begin{aligned} BIC=ln\left\{\frac{1}{nq}||Y-X(X^{\top }X)^{-1}X^{\top }Y\hat{\Delta }(\gamma ,\lambda )||_F^2\right\}+\frac{1}{nq}ln(nq)\cdot p \cdot df(\gamma ,\lambda ), \end{aligned}$$

where $\hat{\Delta }(\gamma , \lambda )$ is the response best-subset selection matrix given $\gamma$ and $\lambda$, and $df(\gamma , \lambda )$ represents the number of nonzero elements in $\hat{\Delta }(\gamma , \lambda )$.

To find the optimal solution for the parameter pair $(\gamma , \lambda )$ minimizing the BIC in (11), we perform a grid search within the two dimensional region of $\gamma \in [0, 1]$ and $\lambda \in \left(min\left\{\frac{{y_j^{\top }}(I-P_X){y_j}}{\left({y_j^{\top }}P_X{y_j}\right)^{1+\gamma }}\right\}-\epsilon ,max\left\{\frac{{y_j^{\top }}(I-P_X){y_j}}{\left({y_j^{\top }}P_X{y_j}\right)^{1+\gamma }}\right\}\right)$, $\epsilon$ is a small positive number. To reduce the computation time, we utilize the following curve search method:

12 $$\begin{aligned} \lambda (\gamma ) = \frac{n-p}{C_{q,p,\alpha }^{1+\gamma }}, \quad \gamma \in [0,1], \end{aligned}$$

where $\alpha$ is the given significance level, $C_{q,p,\alpha }$ is the $\left(1-\frac{\alpha }{q}\right) \times 100\%$ quantile of a specific distribution. We consider it as the inverse of the central $\chi _p^2$ distribution $G_p(\cdot )$ with *p* degrees of freedom, i.e., $C_{q,p,\alpha } = {G_p^{-1}}\left(1-\frac{\alpha }{q}\right).$

## Simulation studies

To evaluate the performance of MRBSS, we compared it with other two existing methods, namely Multivariate linear mixed model (mvLMM) and 2HiGWAS. mvLMM method is powerful approach to detect pleiotropic associations with multiple correlated phenotypes while controlling for population stratification [14]. It considers the genetic effects of a single SNP on multiple phenotypes once a time, and simplifies the parameter estimates required for likelihood ratio test by employing matrix transformation and iteration techniques. 2HiGWAS is a two-stage pleiotropic association analysis method [28]. The first stage is to reduce the model dimension at the sample size using the DC-SIS (distance correlation-based sure independence screening) method, and the second stage is to select the associated SNPs using the grouped penalized regression method.

In simulation studies, the genotypic data of SNPs (take values 0, 1, or 2) are simulated in the R package PhenotypeSimulator [29] with minor allele frequencies equal to 0.4, and the $n \times q$ genotype matrix is denoted as *Y*. The sample size *n* is set to be 100, and the number of SNPs *q* is chosen from $\{100, 200, 500\}$. We generate the $n \times p$ phenotype matrix *X* using the model $Y=X\Theta +\varepsilon$. The number of phenotypes *p* is set to be 5. Assume the proportion of active SNPs to be $q_0$, which is chosen from $\{5\%, 10\%, 20\%\}$. Each row of the first $q \times q_0$ columns of the coefficient matrix $\Theta$ is generated from an uniform distribution, and the elements in the last $q \times (1-q_0)$ columns of the coefficient matrix $\Theta$ are all zeros. Meanwhile, each row of the residual error matrix $\varepsilon$ is generated from a multivariate normal distribution $N(0_q, \Sigma )$. Since the association strengths between pairs of genotype may be various, we consider two kinds of covariance structures for $\Sigma$, which is similarly as those in the recent work of Wang et al. [24]. The first one is the autoregressive structure, i.e., $\Sigma =(\sigma _{kl})_{q\times q}$ with its (*k*, *l*)th element being $\rho ^{|k-l|}$, $(k,l=1,2,\cdots ,q)$. The second one is the compound symmetry structure, i.e., $\Sigma =(1-\rho )I_q+\rho 1_q1_q^{\top }$. We choose $\rho$ from $\{0.2, 0.5, 0.9\}$ to describe the different degree of association strengths. For each of the simulation studies, 1000 repetitions are performed, and the statistical powers and type I error rates are obtained by computing the proportions of *P*-values less than the significance level of 0.05. We evaluate the performance of MRBSS, mvLMM, and 2HiGWAS in terms of statistical power, type I error rate, and computation time.

### Statistical power

**Fig. 1:**
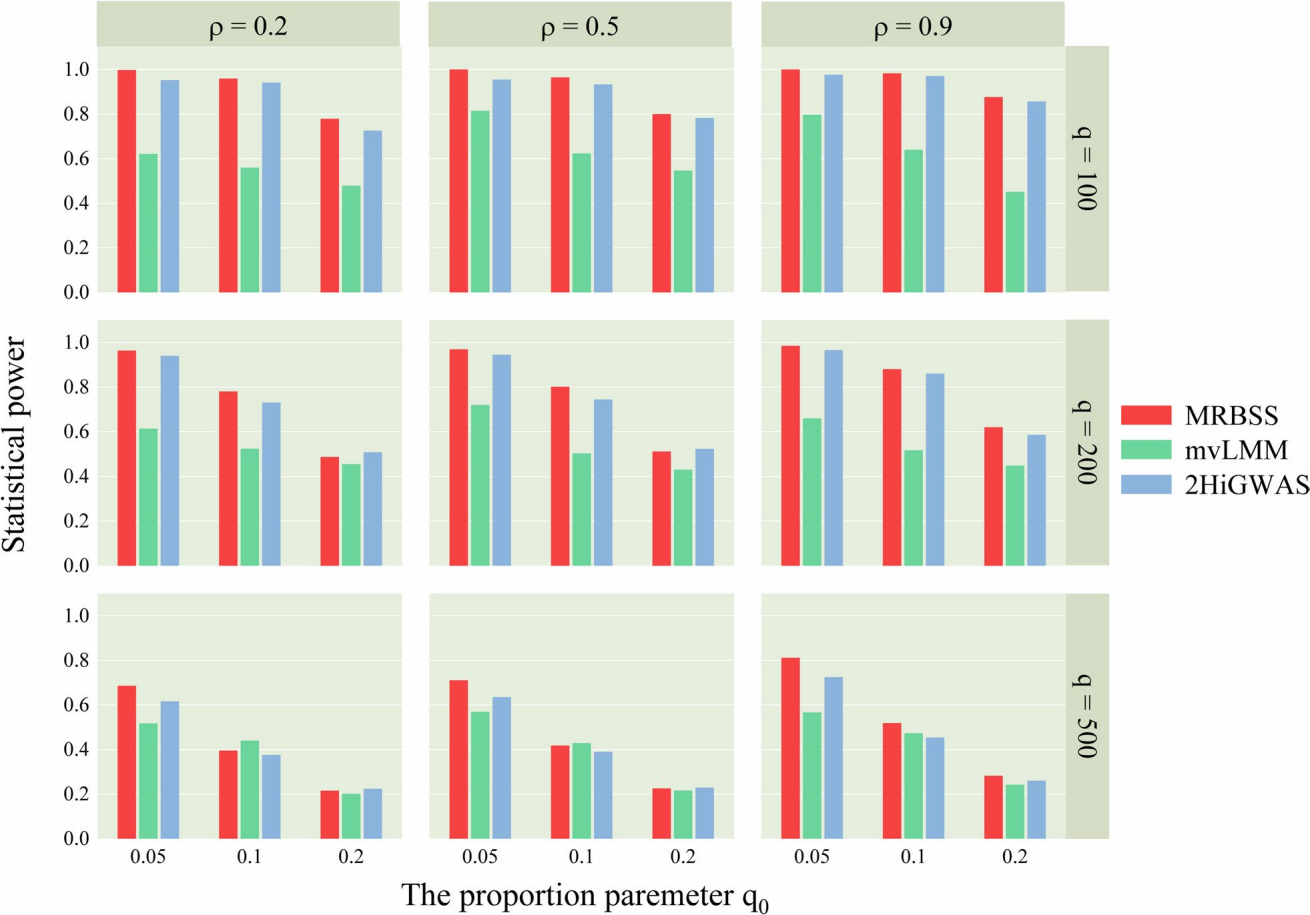
Statistical powers of MRBSS, mvLMM, and 2HiGWAS when $\Sigma$ is of autoregressive structure

**Fig. 2:**
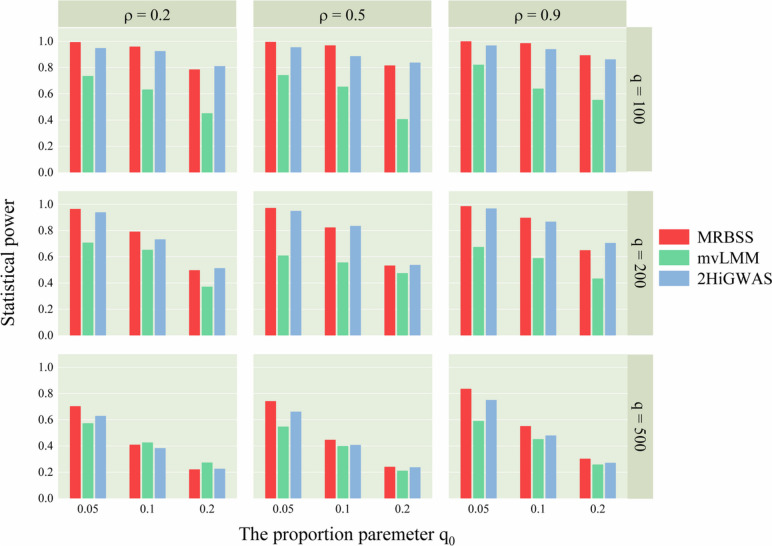
Statistical powers of MRBSS, mvLMM, and 2HiGWAS when $\Sigma$ is of compound-symmetry structure

The statistical power results from nine simulation studies, considering two different covariance structure scenarios, are presented in Figs. 1 and 2.

Figure 1 illustrates that MRBSS exhibits the highest statistical power in most of the considered scenarios. For example, when $(q, \rho ) = (100, 0.2)$ and considering three different proportions of active SNPs ($q_0 = 5\%, 10\%, 20\%$), the powers of MRBSS are 0.9968, 0.9582, and 0.7772, respectively. In comparison, mvLMM achieves powers of 0.6200, 0.5584, and 0.4772, while 2HiGWAS achieves powers of 0.9510, 0.9379, and 0.7243 for the respective proportions. Moreover, the power of MRBSS relative to mvLMM and 2HiGWAS shows minimal change as the association strength $\rho$ increases, indicating its robust performance in detecting association signals with varying strengths. For example, in Fig. 1, with $(q, q_0) = (100, 5\%)$ and different association strengths ($\rho = 0.2, 0.5, 0.9$), the powers of MRBSS are 0.9968, 0.9989, and 0.9991, respectively. In comparison, mvLMM achieves powers of 0.62, 0.8133, and 0.7963, while 2HiGWAS achieves powers of 0.951, 0.9538, and 0.9752 for the corresponding association strengths. Furthermore, as the number of SNPs *q* increases, the statistical powers of all three methods decrease under all scenarios. When the proportion of active SNPs $q_0$ increases to $20\%$, the statistical powers of MRBSS are occasionally lower than those of 2HiGWAS. This observation demonstrates that MRBSS is more suitable for detecting sparse association signals, which are commonly observed in GWAS.

Figure 2 corroborates these findings and yields similar results. The detailed statistical power results under autoregressive structure and compound symmetry structure are showed in Tables S1 and S2, respectively. Notably, the statistical powers under the compound symmetric structure are slightly higher than those under the autoregressive structure. As a result, the proposed MRBSS method consistently exhibits the highest statistical power across the majority of scenarios, enabling it to effectively detect both strong and weak association signals.

### Type I error rate

The type I error rates of the three methods are presented in Tables 1 and 2. Take the results from Table 1 as an example, it is evident that both the MRBSS and mvLMM methods effectively control the type I error rates, as their corresponding *P*-values consistently hover around the predefined significance level of 0.05. Furthermore, the type I error rates of MRBSS and mvLMM demonstrate stability across various scenarios, exhibiting no significant fluctuations with respect to the number of SNPs *q* or the proportions of active SNPs $q_0$. Conversely, the type I error rates of 2HiGWAS significantly surpass the 0.05 threshold, indicating the inflation of false positives. However, as the number of SNPs *q* reaches 500, the type I error rate gradually converges towards 0.05. Similar results can be obtained in Table 2. In summary, both MRBSS and mvLMM demonstrate effective control over the type I error rate in all the scenarios while 2HiGWAS only exhibits favorable performance in scenarios with a large number of SNPs.

**Table 1:** Type I error rates of MRBSS, mvLMM, and 2HiGWAS when $\Sigma$ is of autoregressive structure

*q*	$\rho$	$q_0$	MRBSS	mvLMM	2HiGWAS
100	0.2	5%	0.0698	0.0421	0.2172
		10%	0.0709	0.0333	0.3176
		20%	0.0716	0.075	0.3481
	0.5	5%	0.0701	0.0207	0.2146
		10%	0.0710	0.0378	0.3137
		20%	0.0718	0.0391	0.3468
	0.9	5%	0.0714	0.0506	0.1989
		10%	0.0705	0.0504	0.2963
		20%	0.0720	0.0483	0.3379
200	0.2	5%	0.0654	0.0204	0.1811
		10%	0.0656	0.047	0.1762
		20%	0.0642	0.0497	0.1589
	0.5	5%	0.0652	0.0442	0.1793
		10%	0.0659	0.0562	0.1741
		20%	0.0639	0.0503	0.1554
	0.9	5%	0.0658	0.0497	0.1657
		10%	0.0656	0.0685	0.1648
		20%	0.0645	0.0475	0.1398
500	0.2	5%	0.0623	0.0483	0.0717
		10%	0.0613	0.0653	0.0693
		20%	0.0617	0.055	0.0699
	0.5	5%	0.0625	0.0538	0.0705
		10%	0.0617	0.0212	0.0679
		20%	0.0617	0.0478	0.0687
	0.9	5%	0.0625	0.0284	0.0653
		10%	0.0621	0.0501	0.0610
		20%	0.0615	0.0490	0.0612

**Table 2:** Type I error rates of MRBSS, mvLMM, and 2HiGWAS when $\Sigma$ is of compound-symmetry structure

*q*	$\rho$	$q_0$	MRBSS	mvLMM	2HiGWAS
100	0.2	5%	0.0720	0.0211	0.2138
		10%	0.0733	0.0111	0.3164
		20%	0.0723	0.0125	0.3493
	0.5	5%	0.0721	0.0215	0.2094
		10%	0.0732	0.0781	0.3121
		20%	0.0721	0.0273	0.3453
	0.9	5%	0.0721	0.0433	0.1927
		10%	0.0726	0.0554	0.2887
		20%	0.0722	0.0427	0.3349
200	0.2	5%	0.0654	0.0789	0.1804
		10%	0.0659	0.0389	0.1758
		20%	0.0644	0.0438	0.1576
	0.5	5%	0.0655	0.0332	0.1765
		10%	0.0658	0.0416	0.1726
		20%	0.0648	0.0875	0.1518
	0.9	5%	0.0656	0.0474	0.1615
		10%	0.0662	0.0491	0.1625
		20%	0.0650	0.0527	0.1355
500	0.2	5%	0.0621	0.0425	0.0711
		10%	0.0617	0.0333	0.0687
		20%	0.062	0.0389	0.0695
	0.5	5%	0.0622	0.0561	0.0691
		10%	0.0618	0.0511	0.0661
		20%	0.0621	0.0433	0.0667
	0.9	5%	0.0629	0.0435	0.0637
		10%	0.0618	0.0472	0.0581
		20%	0.0619	0.0479	0.0581

### Computational time

Table 3 presents the average running time (in minutes) of MRBSS, mvLMM, and 2HiGWAS with different association strengths. The detailed running time under autoregressive structure and compound symmetry structure are showed in Tables S3 and S4, respectively. It is evident that MRBSS offers a clear computational advantage over 2HiGWAS, followed by mvLMM in all scenarios. For example, when $q=100$ and considering different proportions of active SNPs ($q_0=5\%, 10\%, 20\%$), the average computation times of MRBSS are 32.16, 32.22, and 29.37 minutes, respectively. In comparison, 2HiGWAS requires significantly longer computation times of 108.23, 108.81, and 107.48 minutes, respectively, which are more than three times longer than those of MRBSS. mvLMM exhibits the longest computation times, with values of 159.34, 156.53, and 164.22 minutes, respectively, which are nearly five times longer than those of MRBSS. Consequently, the proposed MRBSS method significantly reduces the computational burden, providing a notable advantage in terms of efficiency compared to mvLMM and 2HiGWAS.

**Table 3:** The average running time (minutes) of MRBSS, mvLMM, and 2HiGWAS for 1000 repetitions

Method		MRBSS			mvLMM			2HiGWAS	
$q_0$	5%	10%	20%	5%	10%	20%	5%	10%	20%
$q=100$	32.16	32.22	29.37	159.34	156.53	164.22	108.23	108.81	107.48
$q=200$	154.79	155.39	159.44	253.39	252.19	266.78	233.23	233.78	238.54
$q=500$	270.48	268.42	237.09	887.61	862.93	917.92	639.05	637.36	637.51

## Real data applications

### Application to maize yield-related traits datasets

Corn is a globally significant food crop, serving as both animal feed and an industrial raw material. The yield-related traits of corn play a direct role in determining its final production. Therefore, investigating the shared genetic factors underlying these yield traits is crucial for achieving high crop yields. In this study, seven maize yield-related traits are applied for analysis, including ear length (EL), ear diameter (ED), cob diameter (CD), kernel number per row (KNPR), 100 grain weight (100-GW), cob weight (CW), and kernel width (KW) [30]. Moreover, missing phenotypic values are imputed using the mean and subsequently standardized, SNPs with a minor allele frequency below 0.05 are removed. Finally, 368 samples and 557,893 SNPs are remained for the seven yield-related traits.

**Fig. 3:**
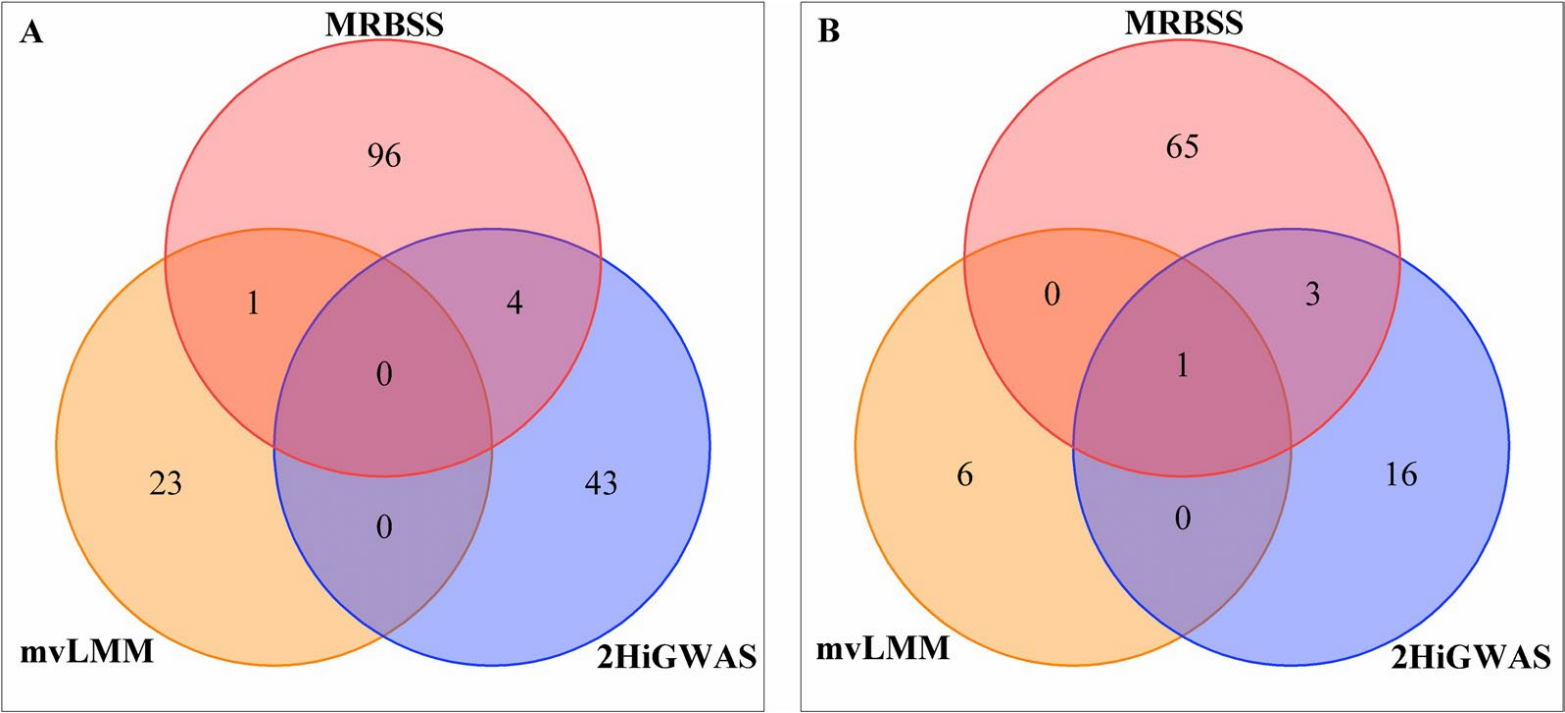
The venn diagram for the overlapped genes identified by MRBSS, mvLMM, and 2HiGWAS. **A** in maize yield-related traits; (**B**) in pig blood lipid traits

We use the proposed MRBSS method, along with two other methods, namely mvLMM and 2HiGWAS, to conduct pleiotropic genetic association analyse on seven maize yield-related traits. The results reveal a total of 151, 30, and 66 significantly associated SNPs detected by MRBSS, mvLMM, and 2HiGWAS, respectively. To ascertain the corresponding mapped genes for these associated SNPs, we refer to the B73 RefGENV4 genome in the maize databases available at MaizeGDB (www.maizegdb.org). By searching within a 200kb range upstream and downstream of the SNPs, we identify a count of 101, 24, and 47 associated genes using the aforementioned methods, respectively. Moreover, we observed one gene that is identified by both MRBSS and mvLMM, as well as four genes that are identified by both MRBSS and 2HiGWAS. The Venn diagram (Fig. 3(A)) shows the overlapping genes detected by the three methods in maize yield traits. Therefore, we can conclude that MRBSS identifies more pleiotropic associations for maize yield-related traits. Moreover, to assess the prior knowledge regarding the identified associated genes, we conduct an extensive investigation in the NCBI Gene database (www.ncbi.nlm.nih.gov) as well as literature repositories such as PubMed. The findings reveal that out of the genes identified by the MRBSS method, eight have previously been reported to be associated with maize yield-related traits. Similarly, one gene detected by the mvLMM method has been reported in previous studies. In contrast, none of the genes identified by the 2HiGWAS method have been previously reported. We supply a comprehensive overview of the identified genes and their corresponding information in Table S5.

### Application to pig lipid traits datasets

Blood lipids are ubiquitously present in the cellular milieu of animals, and they play essential roles in fundamental metabolic processes. Their intricate involvement in the pathogenesis of cardiovascular diseases, obesity, metabolic syndrome, and diabetes has been extensively documented [31]. In this study, we focus on the blood lipid traits of the Laiwu pig [32], which is an indigenous Chinese breed. A dataset comprising 316 specimens and 61,565 SNPs is collected for six blood lipid traits, including total cholesterol (TC), triglycerides (TG), high-density lipoprotein cholesterol (HDL), low-density lipoprotein cholesterol (LDL), HDL-C/LDL-C ratio, and atherosclerosis index (AI). Missing phenotypic values are imputed using the mean and subsequently standardized.

Similarly, we employ three distinct methods, namely MRBSS, mvLMM, and 2HiGWAS, to perform pleiotropic genetic association analyse on the six blood lipid phenotypes of the Laiwu pig. Through these analyses, we identify a total of 121, 8, and 41 SNPs significantly associated with the blood lipid traits using MRBSS, mvLMM, and 2HiGWAS, respectively. Assuming that the mapped genes are located within a 20kb range upstream and downstream of the associated SNPs, we observe a count of 69, 7, and 20 genes for the three methods, respectively. Furthermore, one gene is identified as associated by all three methods, and three genes are detected by both MRBSS and 2HiGWAS. To visualize the overlapping genes detected by the three methods in pig lipid traits, a Venn diagram (Fig. 3(B)) is constructed. Consequently, we can conclude that MRBSS exhibits a greater capacity to identify pleiotropic associations for pig lipid traits.

Additionally, through a comprehensive search of various databases, including those available in the literature, we discover that eight genes identified by MRBSS, four genes identified by mvLMM, and two genes identified by 2HiGWAS have been previously reported. Further information on the identified genes, along with their corresponding details, can be found in Table S6.

## Discussion

Pleiotropic genetic association analysis offers the potential to uncover complex relationships between genetic variants and multiple phenotypes. Nowadays, it is a hot topic to develop the statistical methodologies for it. In this study, we propose to use MRBSS method to detect pleiotropic associations. Our findings demonstrate that MRBSS has a high power for identifying association signals with varying strengths and different correlation structures. This highlights the versatility and robustness of MRBSS in capturing genetic associations in complex phenotypes. Moreover, MRBSS provides effective control over the type I error rate in all the considered scenarios, ensuring a low level of false positive results. In terms of computational efficiency, MRBSS shows the shortest running time.

The good performance of the MRBSS method can be attributed to the following three aspects: First, in the construction of the multivariate response variable regression genetic model, all phenotypes are considered as predictor variables, taking into account the complex hidden correlation information among phenotypes. Second, SNPs are treated as response variables, and the response best-subset selection approach considers both the inherent interactions among SNPs and avoids multiple testing corrections. Third, by transforming the response variable selection procedure into a pure 0-1 integer optimization problem, redundant SNPs are removed. Parameter estimation is performed using the curve search method, thereby reducing computational complexity.

In fact, the mvLMM used for comparison is a typical multi-trait single-locus association analysis method. As the number of phenotypes increases, its computational complexity increases dramatically [14]. 2HiGWAS is a multi-trait multi-locus association analysis method commonly used for longitudinal analysis of phenotypes that change over time [28]. From our simulation results, it can be observed that when the signals are sparse ($q_0=5\%, 10\%$), it does not have high statistical power. However, as the signals become dense ($q_0=20\%$), its statistical power can sometimes even exceed that of MRBSS. Additionally, as the number of SNPs increases, the type I error rate of 2HiGWAS is controlled at a reasonable level. These findings indicate that 2HiGWAS is more suitable for detecting dense signals in high-dimensional data.

Although MRBSS is developed for pleiotropic genetic association analysis, it can also be extended to other areas, such as association analysis on longitudinal phenotypes and transcriptome-wide association study, where the association between longitudinal phenotypes and genetic variants, the association between gene expression levels and genetic variants, are performed, respectively. MRBSS will contribute to a deeper understanding of the genetic basis of complex phenotypes and diseases. However, MRBSS has weak ability to explain the genetic effects of the pleiotropic genetic associations, further studies would be focus on addressing this tissue.

## Conclusion

In summary, we propose an efficient pleiotropic genetic association analysis method based on multivariate response best-subset selection, which not only considers the correlation structure in multiple phenotypes but also the internal interaction effect between multiple loci. Simulation experiments show that the method remarkably reduces the computational time, obtains higher statistical power under most of the considered scenarios, and controls the type I error rate at a low level. The application studies in the datasets of maize yield-related traits and pig lipid traits further verifies the effectiveness.

### Supplementary Information

**Additional file 1.** **Tables S1-S6** are showed in the Supplementary Materials.

## Data Availability

The procedures to generate the simulated data have been described in the manuscript. The maize data is freely available online at http://www.maizego.org/Resources.html, and the pig data is freely available online at https://datadryad.org/stash/dataset/doi:10.5061/dryad.4gh70.
