# Supplementary material for: Pleiotropic genetic association analysis with multiple phenotypes using multivariate response best-subset selection
Source: BMC Genomics. 2023 Dec 11;24:759. doi: 10.1186/s12864-023-09820-5 (PMC10712198; doi:10.1186/s12864-023-09820-5)
Supplement: Supplementary file 1 — Additional file 1. Tables S1-S6 are showed in the Supplementary Materials. [file 12864_2023_9820_MOESM1_ESM.pdf]

Supplement Materials for "Pleiotropic genetic association analysis with multiple phenotypes using multivariate response best-subset selection"

Table S1: Statistical powers of MRBSS, mvLMM, and 2HiGWAS when Σ is of autoregressive structure.

q	ρ	q_0	MRBSS	mvLMM	2HiGWAS
100	0.2	5%	0.9968	0.62	0.951
		10%	0.9582	0.5584	0.9397
		20%	0.7772	0.4772	0.7243
	0.5	5%	0.9989	0.8133	0.9538
		10%	0.9636	0.6217	0.9321
		20%	0.7989	0.5451	0.7815
	0.9	5%	0.9991	0.7963	0.9752
		10%	0.9819	0.6389	0.9698
		20%	0.8754	0.4504	0.8553
200	0.2	5%	0.9627	0.6125	0.939
		10%	0.7789	0.5221	0.7293
		20%	0.4851	0.4532	0.5066
	0.5	5%	0.9684	0.7186	0.944
		10%	0.8	0.5011	0.7431
		20%	0.5095	0.4279	0.5211
	0.9	5%	0.9835	0.6583	0.9645
		10%	0.8795	0.5152	0.8592
		20%	0.6184	0.4463	0.5841
500	0.2	5%	0.6847	0.516	0.6149
		10%	0.3938	0.4389	0.3744
		20%	0.2142	0.2	0.2229
	0.5	5%	0.7098	0.5676	0.6342
		10%	0.4162	0.4279	0.3888
		20%	0.2242	0.2148	0.2279
	0.9	5%	0.8102	0.5655	0.7240
		10%	0.5173	0.4718	0.4524
		20%	0.2811	0.2413	0.2590

Table S2: Statistical powers of MRBSS, mvLMM, and 2HiGWAS when Σ is of compound-symmetry structure.

q	ρ	q_0	MRBSS	mvLMM	2HiGWAS
100	0.2	5%	0.993	0.7344	0.9472
		10%	0.9583	0.631	0.9244
		20%	0.7843	0.45	0.8096
	0.5	5%	0.9949	0.7412	0.9534
		10%	0.9678	0.6531	0.8856
		20%	0.8151	0.4056	0.8364
	0.9	5%	0.9992	0.8207	0.9674
		10%	0.9847	0.6381	0.9394
		20%	0.8928	0.5519	0.8617
200	0.2	5%	0.9635	0.7063	0.9382
		10%	0.7908	0.6519	0.7315
		20%	0.4961	0.3707	0.5117
	0.5	5%	0.9713	0.6085	0.9486
		10%	0.8225	0.5550	0.8342
		20%	0.5317	0.4739	0.5365
	0.9	5%	0.9849	0.6736	0.9671
		10%	0.8961	0.5885	0.8662
		20%	0.6484	0.4328	0.7038
500	0.2	5%	0.7019	0.5722	0.6284
		10%	0.4084	0.425	0.3825
		20%	0.2197	0.2721	0.2246
	0.5	5%	0.7408	0.5463	0.6604
		10%	0.4455	0.3982	0.4066
		20%	0.2396	0.21	0.2361
	0.9	5%	0.8345	0.5897	0.7496
		10%	0.5506	0.4505	0.4786
		20%	0.3013	0.2572	0.2700

Table S3: The running time (minutes) of MRBSS, mvLMM, and 2HiGWAS when Σ is of autoregressive structure.

q	ρ	q_0	MRBSS	mvLMM	2HiGWAS
100	0.2	5%	32.54	163.56	107.05
		10%	30.64	158.73	110.43
		20%	27.13	156.34	105.82
	0.5	5%	32.13	157.26	108.16
		10%	33.78	164.35	107.19
		20%	30.37	167.30	108.75
	0.9	5%	31.83	157.20	109.46
		10%	32.25	146.52	108.82
		20%	30.61	169.03	107.88
200	0.2	5%	140.62	203.32	238.46
		10%	133.46	221.63	232.82
		20%	133.17	248.7	236.65
	0.5	5%	152.06	354.28	226.74
		10%	161.55	278.37	231.79
		20%	183.04	312.43	239.35
	0.9	5%	171.71	202.29	234.51
		10%	171.16	256.58	236.74
		20%	162.11	239.21	239.62
500	0.2	5%	879.32	854.91	910.75
		10%	776.71	878.83	906.01
		20%	772.18	905.89	909
	0.5	5%	849.06	895.62	632.55
		10%	848.66	892.05	641.93
		20%	854.79	923.20	639.61
	0.9	5%	270.48	887.61	639.05
		10%	268.42	862.93	637.36
		20%	237.09	917.82	637.51

Table S4: The running time (minutes) of MRBSS, mvLMM, and 2HiGWAS when Σ is of compound-symmetry structure.

q	ρ	q_0	MRBSS	mvLMM	2HiGWAS
100	0.2	5%	28.48	251.33	99.49
		10%	27.45	247.54	98.24
		20%	27.32	240.66	100.35
	0.5	5%	31.17	283.43	80.07
		10%	30.63	277.36	84.23
		20%	29.82	313.84	82.92
	0.9	5%	29.87	251.69	78.89
		10%	29.36	247.57	90.65
		20%	30.18	263.52	97.68
200	100	5%	164.76	343.61	283.77
		10%	165.95	328.24	278.79
		20%	166.51	332.96	287.82
	0.5	5%	212.24	346.87	236.76
		10%	217.37	358.43	230.38
		20%	215.71	350.96	230.49
	0.9	5%	217.04	341.38	233.55
		10%	212.18	363.90	233.72
		20%	214.89	358.48	232.73
500	0.2	5%	532.59	889.26	947.79
		10%	551.88	911.82	944.47
		20%	527.03	890.15	952.24
	0.5	5%	519.26	1080.14	624.44
		10%	524.47	973.16	626.02
		20%	530.29	984.07	632.53
	0.9	5%	544.88	923.42	947.85
		10%	538.86	908.54	951.22
		20%	540.78	937.48	947.59

Table S5: The comprehensive overview of the identified genes of MRBSS, mvLMM, and 2HiGWAS in maize yield-related traits.

Method	chr	Position	SNP	gene	PMID	Publish Information
MRBSS	chr2	193419820	S_69547729	TRNAY-GUA		
	chr9	120363553	S_6408654	TRNAS-GCU		
	chr2	170917974	S_69547729	TRNAR-CCG		
	chr4	5379900	S_69547729	TRNAL-UAA		
	chr1	8679773	S_79867645	**TRNAK-UUU**		
	chr8	502662	S_6408654	ms23	27913638	Nan et al. 2017 [1]
	chr2	1680098	S_69547729	LOC100284690		
	chr1	161992018	S_69547729	LOC732793	29089394	Salesse et al. 2017 [2]
	chr7	19053889	S_79867645	LOC606461	15916952	Hamant et al. 2005 [3]
	chr3	199269825	S_69547729	LOC542740	11080288	McGonigle et al. 2000 [4]
	chr9	149820221	S_136912398	**LOC542502**	15020742	Gardiner et al. 2004 [5]
	chr1	287718408	S_69547729	LOC542099	15466291	Lai et al. 2004 [6]
	chr4	30925830	S_69547729	LOC542010	31399756	Gao et al. 2019 [7]
	chr9	152087076	S_136912398	LOC541639	19580677	Vega-Arreguin et al. 2009 [8]
	chr4	124931226	S_69547729	LOC118476944		
	chr1	239508615	S_69547729	LOC118476141		
	chr7	175832688	S_175832688	LOC100276089		
	chr3	220682851	S_69547729	**LOC100276047**		
	chr4	124931226	S_69547729	LOC118476944		
	chr1	239508615	S_69547729	**LOC118476141**		
mvLMM	chr1	76682243	S_231963909	TRNAT-UGU		
	chr9	149820221	S_28612058	**LOC542502**	15020742	Gardiner et al. 2004 [5]
	chr6	109600304	S_231963909	LOC118472228		
	chr5	216083126	S_231963909	LOC118472114		
	chr5	3548861	S_231963909	LOC118471978		
	chr5	77434100	S_231963909	LOC111589387		
	chr1	65745651	S_65745651	LOC109943636		
	chr6	83930639	S_231963909	LOC109940320		
	chr4	233827929	S_231963909	LOC103655965		
	chr4	151655102	S_26394568	LOC103653831		
	chr3	147591060	S_65745651	LOC103650695		
	chr3	47964061	S_65745651	LOC103650045		
	chr1	297376856	S_65745651	LOC103644212		
	chr3	167446685	S_167446685	LOC103643102		

Continued on next page

Continued

Method	chr	Position	SNP	gene	PMID	Publish Information
	chr8	8288060	S_28612058	LOC103634732		
	chr7	170321339	S_28612058	LOC103633361		
	chr7	124923304	S_231963909	LOC103632796		
	chr5	193967137	S_231963909	LOC103628775		
	chr2	2115327	S_65745651	LOC100501244		
	chr3	216528556	S_9043814	LOC100383722		
2HiGWAS	chr8	172759352	S_102947394	TRNAQ-UUG		
	chr1	8679773	S_190563430	**TRNAK-UUU**		
	chr1	239508615	S_11952220	**LOC118476141**		
	chr3	182165625	S_11952220	LOC111591215		
	chr5	60801763	S_11952220	LOC111589268		
	chr3	205375124	S_11952220	LOC109945325		
	chr3	145741985	S_11952220	LOC109945290		
	chr4	2655193	S_11952220	LOC109945367		
	chr1	11952220	S_11952220	LOC103631736		
	chr6	138248016	S_190563430	LOC103630069		
	chr6	106340331	S_190563430	LOC103629702		
	chr6	100205456	S_190563430	LOC103629632		
	chr6	44339590	S_190563430	LOC103629240		
	chr5	174409902	S_218533121	LOC103627265		
	chr5	145510183	S_145510183	LOC103626988		
	chr5	80971305	S_11952220	LOC103626608		
	chr5	58752703	S_11952220	LOC103626405		
	chr3	220682851	S_11952220	**LOC100276047**		
	chr1	244771872	S_11952220	LOC100273083		

* The top 20 identified genes are listed in the table, and the bold parts are published genes.

Table S6: The comprehensive overview of the identified genes of MRBSS, mvLMM, and 2HiGWAS in pig lipid traits.

Method	chr	Position	SNP	gene	PMID	Publish Information
	chr16	35409659	ALGA0090178	ANKRD55		
	chr9	12069732	M1GA0012740	AQP11	27646534	Li et al. 2017 [9]
	chr1	133696123	M1GA0023456	AQP9	27646534	Li et al. 2017 [9]
	chr16	33577312	ALGA0090097	ARL15		
	chr10	51997012	ASGA0048126	ARMC3		
	chr17	41375991	ALGA0094774	BPI	23661399	Zhu et al. 2013 [10]
	chr1	134378501	INRA0004022	C1H15orf41		
	chr18	43229113	ASGA0089892	CHN2		
	chr7	116227002	ASGA0036012	DICER1	26433267	Knapczyk-Stwora et al. 2015 [11]
MRBSS	chr15	33206322	ASGA0090518	DLGAP2		
	chr13	31398134	ALGA0115724	IP6K2		
	chr12	16710957	ALGA0110097	**ITGB3**	12030937	Sanz et al. 2002 [12]
	chr2	118604464	H3GA0007383	**KCNN2**		
	chr1	113553257	MARC0041267	LIPC	21327595	Jiang et al. 2011 [13]
	chr9	11519204	ASGA0041513	LOC100525597		
	chr4	38445819	ASGA0019342	LOC102163076		
	chr2	44728053	ASGA0100252	PSMA1	23550158	Miles et al. 2013 [14]
	chr9	102585036	SIRI0000798	PTPN12		
	chr16	37423396	M1GA0020993	**RAB3C**		
	chr3	60254948	ALGA0114282	SUCLG1	26249701	Fraser et al. 2015 [15]
	chr5	96953817	ALGA0033594	LRRIQ1		
	chr6	77639059	ALGA0121267	UBR4		
	chr9	10050483	MARC0068884	DGAT2	26407871	Zang et al. 2016 [16]
mvLMM	chr11	20304315	ASGA0050154	HTR2A	29104045	Lim et al. 2016 [17]
	chr12	13082107	DRGA0011623	PRKCA	24058600	Zhang et al. 2013 [18]
	chr16	37423396	M1GA0020993	**RAB3C**		
	chr15	92403979	INRA0049812	TFPI	18611227	Lee et al. 2008 [19]
	chr13	91193838	DRGA0012647	CLRN1		
	chr14	59708910	ALGA0077781	COG2		
	chr23	8673401	CAHM0000169	FRMPD4		
	chr8	35774390	ASGA0090208	GNPDA2	15679890	Jorgensen et al. 2005 [20]
	chr12	16710957	ALGA0110097	**ITGB3**	12030937	Sanz et al. 2002 [12]
	chr2	118658359	ALGA0015125	**KCNN2**		
	chr1	52520680	DRGA0000780	KCNQ5		

Continued on next page

Continued

Method	chr	Position	SNP	gene	PMID	Publish Information
	chr3	117566357	MARC0008715	LDAH		
	chr2	29658099	H3GA0006435	LOC100517025		
2HiGWAS	chr12	60015994	ASGA0100102	LOC102157639		
	chr11	5077000	MARC0094302	LOC106505238		
	chr3	101451356	ASGA0104226	MORN2		
	chr23	20627071	MARC0011285	PCYT1B		
	chr16	37423396	M1GA0020993	**RAB3C**		
	chr2	89499964	ASGA0010781	RASGRF2		
	chr4	2495035	ASGA0017090	SLC45A4		
	chr3	17668241	M1GA0004123	SRCAP		
	chr16	56305607	ALGA0090761	TENM2		
	chr24	953841	CAHM0000184	TRAF2		
	chr15	31995248	ALGA0123902	TUBGCP5		

* The top 20 identified genes are listed in the table, and the bold parts are published genes.

References

[1] Nan, G. L., Zhai, J., Arikit, S., et al. (2017). MS23, a master basic helix-loop-helix factor, regulates the specification and development of the tapetum in maize. *Development*. 144(1): 163-172.

[2] Salesse, C., Sharwood, R., Sakamoto, W., et al. (2017). The rubisco chaperone BSD2 may regulate chloroplast coverage in maize bundle sheath cells. *Plant Physiology*. 175(4): 1624-1633.

[3] Hamant, O., Golubovskaya, I., Meeley, R., (2005). A REC8-dependent plant Shugoshin is required for maintenance of centromeric cohesion during meiosis and has no mitotic functions. *Current Biology*. 15(10): 948-954.

[4] McGonigle, B., Keeler, S. J., Lau, S. M., et al. (2000). A genomics approach to the comprehensive analysis of the glutathione S-transferase gene family in soybean and maize. *Plant Physiology*. 124(3): 1105-1120.

[5] Gardiner, J., Schroeder, S., Polacco, M. L., et al. (2004). Anchoring 9,371 maize expressed sequence tagged unigenes to the bacterial artificial chromosome contig map by two-dimensional overgo hybridization. *Plant Physiology*. 134(4):1317-1326.

[6] Lai, J., Dey, N., Kim, C. S., et al. (2004). Characterization of the maize endosperm transcriptome and its comparison to the rice genome. *Genome Research*. 14(10A): 1932-1937.

[7] Gao, L., Yang, G., Li, Y., et al. (2019). Fine mapping and candidate gene analysis of a QTL associated with leaf rolling index on chromosome 4 of maize (Zea mays L.). *Theoretical and Applied Genetics.* 132(11): 3047-3062.

[8] Vega-Arregun, J. C., Ibarra-Laclette, E., Jimnez-Moraila, B., et al. (2009). Deep sampling of the Palomero maize transcriptome by a high throughput strategy of pyrosequencing. *BMC Genomics.* 10: 299.

[9] Li, M., Chen, L., Tian, S., et al. (2017). Comprehensive variation discovery and recovery of missing sequence in the pig genome using multiple de novo assemblies. *Genome Research.* 27(5): 865-874.

[10] Zhu, J., Zi, C., Wu, Z. C., et al. (2013). Age-dependent expression of the BPI gene in Sutai piglets. *Genetics and Molecular Research.* 12(2): 2120-2126.

[11] Knapczyk-Stwora, K., Belej, A., Grzesiak, M., et al. (2015). Effect of gestational antiandrogen treatment on Dicer1 expression in the porcine fetal gonads. *Acta Histochem.* 117(8): 725-731.

[12] Sanz, L. M., Jimnez-Marn, A., Yerle, M., et al. (2002). A polymorphic microsatellite located on pig chromosome band 12p11-2/3p13, within the 3'-UTR of the ITGB3 gene. *Animal Genetics.* 33(3): 239-240.

[13] Jiang, C., Liu, Y. (2011). A novel porcine gene, LIPC, differentially expressed in the liver tissues from Meishan and Large White pigs. *Journal of Applied Genetics.* 52(2): 219-223.

[14] Miles, E. L., O'Gorman, C., Zhao, J., et al. (2013). Transgenic pig carrying green fluorescent proteasomes. *Proceedings of the National Academy of Sciences.* 110(16): 6334-6339.

[15] Fraser, M. E., Hayakawa, K., Hume, M. S., et al. (2006). Interactions of GTP with the ATP-grasp domain of GTP-specific succinyl-CoA synthetase. *Journal of Biological Chemistry.* 281(16):11058-11065.

[16] Zang, L., Wang, Y., Sun, B., et al. (2016). Identification of a 13 bp indel polymorphism in the 3'-UTR of DGAT2 gene associated with backfat thickness and lean percentage in pigs. *Gene.* 576(2): 729-733.

[17] Lim, I., Chess-Williams, R., Sellers, D. (2018). 5-HT2A receptor is the predominant receptor mediating contraction of the isolated porcine distal ureter to 5-HT in young and old animals. *European Journal of Pharmacology.* 818: 328-334.

[18] Zhang, Y., Hermanson, M. E., Eddinger, T. J. (2013). Tonic and phasic smooth muscle contraction is not regulated by the PKC-CPI-17 pathway in swine stomach antrum and fundus. *PLoS One.* 8(9): e74608.

[19] Lee, K. F., Salvaris, E. J., Roussel, J. C., et al. (2008). Recombinant pig TFPI efficiently regulates human tissue factor pathways. *Xenotransplantation.* 15(3): 191-197.

[20] Jorgensen, F. G., Hobolth, A., Hornshoj, H., et al. (2005). Comparative analysis of protein coding sequences from human, mouse and the domesticated pig. *BMC Biology.* 28(3): 2.
